# The Structural Basis of Coenzyme A Recycling in a Bacterial Organelle

**DOI:** 10.1371/journal.pbio.1002399

**Published:** 2016-03-09

**Authors:** Onur Erbilgin, Markus Sutter, Cheryl A. Kerfeld

**Affiliations:** 1 Department of Plant and Microbial Biology, University of California, Berkeley, Berkeley, California, United States of America; 2 MSU-DOE Plant Research Laboratory and Department of Biochemistry and Molecular Biology, Michigan State University, East Lansing, Michigan, United States of America; 3 Molecular Biophysics and Integrated Bioimaging Division, Lawrence Berkeley National Laboratory, Berkeley, California, United States of America; Brandeis University, UNITED STATES

## Abstract

Bacterial Microcompartments (BMCs) are proteinaceous organelles that encapsulate critical segments of autotrophic and heterotrophic metabolic pathways; they are functionally diverse and are found across 23 different phyla. The majority of catabolic BMCs (metabolosomes) compartmentalize a common core of enzymes to metabolize compounds via a toxic and/or volatile aldehyde intermediate. The core enzyme phosphotransacylase (PTAC) recycles Coenzyme A and generates an acyl phosphate that can serve as an energy source. The PTAC predominantly associated with metabolosomes (PduL) has no sequence homology to the PTAC ubiquitous among fermentative bacteria (Pta). Here, we report two high-resolution PduL crystal structures with bound substrates. The PduL fold is unrelated to that of Pta; it contains a dimetal active site involved in a catalytic mechanism distinct from that of the housekeeping PTAC. Accordingly, PduL and Pta exemplify functional, but not structural, convergent evolution. The PduL structure, in the context of the catalytic core, completes our understanding of the structural basis of cofactor recycling in the metabolosome lumen.

## Abstract 2

This study describes the structure of a novel phosphotransacylase enzyme that facilitates the recycling of the essential cofactor acetyl-CoA within a bacterial organelle and discusses the properties of the enzyme's active site and how it is packaged into the organelle.

## Author Summary

In metabolism, molecules with “high-energy” bonds (e.g., ATP and Acetyl~CoA) are critical for both catabolic and anabolic processes. Accordingly, the retention of these bonds during biochemical transformations is incredibly important. The phosphotransacylase (Pta) enzyme catalyzes the conversion between acyl-CoA and acyl-phosphate. This reaction directly links an acyl-CoA with ATP generation via substrate-level phosphorylation, producing short-chain fatty acids (e.g., acetate), and also provides a path for short-chain fatty acids to enter central metabolism. Due to this key function, Pta is conserved across the bacterial kingdom. Recently, a new type of phosphotransacylase was described that shares no evolutionary relation to Pta. This enzyme, PduL, is exclusively associated with organelles called bacterial microcompartments, which are used to catabolize various compounds. Not only does PduL facilitate substrate level phosphorylation, but it also is critical for cofactor recycling within, and product efflux from, the organelle. We solved the structure of this convergent phosphotransacylase and show that it is completely structurally different from Pta, including its active site architecture. We also discuss features of the protein important to its packaging in the organelle.

## Introduction

Bacterial Microcompartments (BMCs) are organelles that encapsulate enzymes for sequential biochemical reactions within a protein shell [1–4]. The shell is typically composed of three types of protein subunits, which form either hexagonal (BMC-H and BMC-T) or pentagonal (BMC-P) tiles that assemble into a polyhedral shell. The facets of the shell are composed primarily of hexamers that are typically perforated by pores lined with highly conserved, polar residues [1] that presumably function as the conduits for metabolites into and out of the shell [5,6].

**Fig 1:**
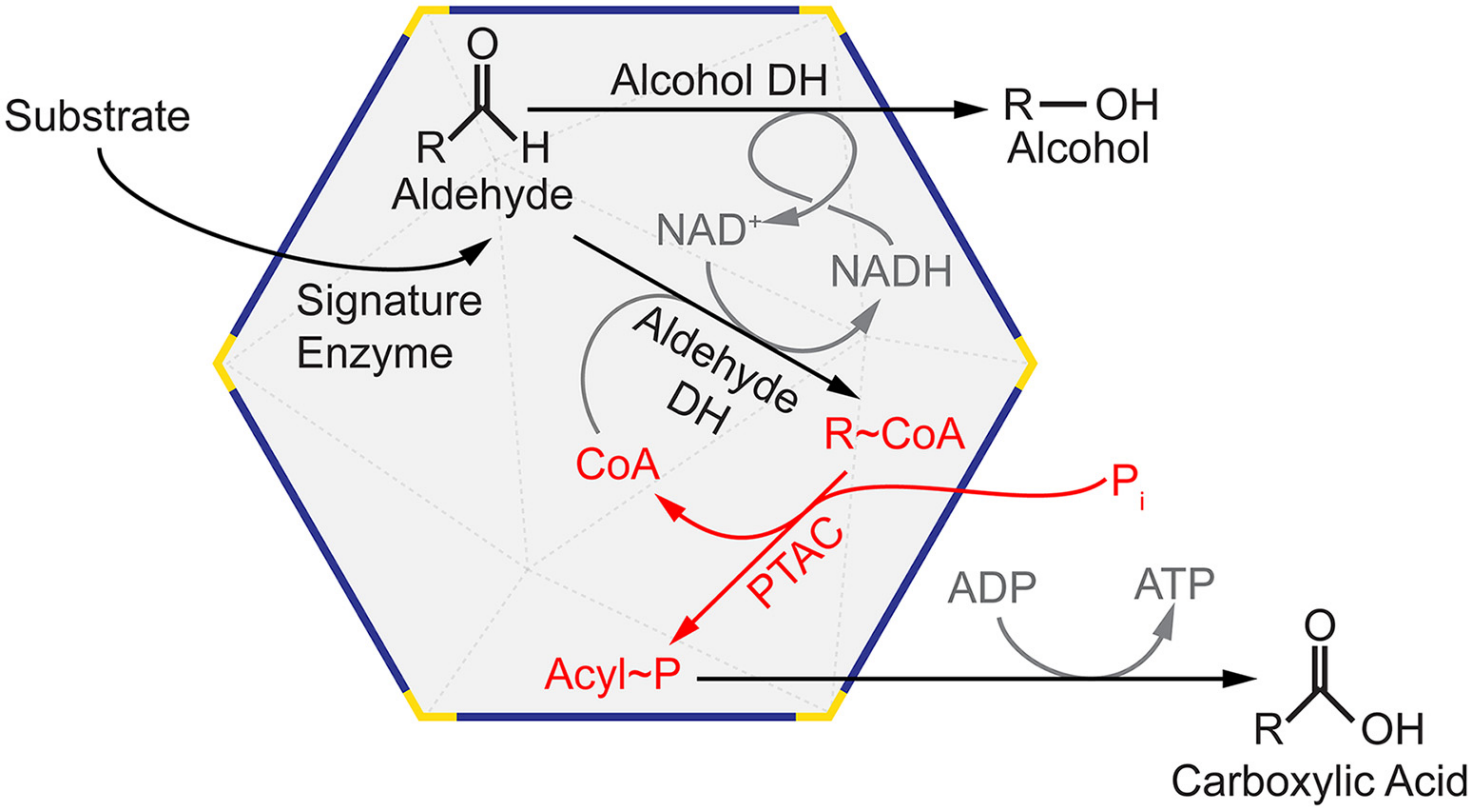
General biochemical model of aldehyde-degrading BMCs (metabolosomes) illustrating the common metabolosome core enzymes and reactions. Substrates and cofactors involving the PTAC reaction are shown in red; other substrates and enzymes are shown in black, and other cofactors are shown in gray.

The vitamin B_12_-dependent propanediol-utilizing (PDU) BMC was one of the first functionally characterized catabolic BMCs [7]; subsequently, other types have been implicated in the degradation of ethanolamine, choline, fucose, rhamnose, and ethanol, all of which produce different aldehyde intermediates (Table 1). More recently, bioinformatic studies have demonstrated the widespread distribution of BMCs among diverse bacterial phyla [2,8,9] and grouped them into 23 different functional types [2]. The reactions carried out in the majority of catabolic BMCs (also known as metabolosomes) fit a generalized biochemical paradigm for the oxidation of aldehydes (Fig 1) [2]. This involves a BMC-encapsulated signature enzyme that generates a toxic and/or volatile aldehyde that the BMC shell sequesters from the cytosol [1]. The aldehyde is subsequently converted into an acyl-CoA by aldehyde dehydrogenase, which uses NAD^+^ and CoA as cofactors [10,11]. These two cofactors are relatively large, and their diffusion across the protein shell is thought to be restricted, necessitating their regeneration within the BMC lumen [3,12,13]. NAD^+^ is recycled via alcohol dehydrogenase [13], and CoA is recycled via phosphotransacetylase (PTAC) [3,12] (Fig 1). The final product of the BMC, an acyl-phosphate, can then be used to generate ATP via acyl kinase, or revert back to acyl-CoA by Pta [14] for biosynthesis. Collectively, the aldehyde and alcohol dehydrogenases, as well as the PTAC, constitute the common metabolosome core.

**Table 1:** Characterized and predicted catabolic BMC (metabolosome) types that represent the aldehyde-degrading paradigm (for definition of types see Kerfeld and Erbilgin [1]).

Name	PTAC Type	Sequestered Aldehyde
PDU*	PduL	propionaldehyde
EUT1	PTA_PTB	acetaldehyde
EUT2	PduL	acetaldehyde
ETU	None	acetaldehyde
GRM1/CUT	PduL	acetaldehyde
GRM2	PduL	acetaldehyde
GRM3*,4	PduL	propionaldehyde
GRM5/GRP	PduL	propionaldehyde
PVM*	PduL	lactaldehyde
RMM1,2	None	unknown
SPU	PduL	unknown

* PduL from these functional types of metabolosomes were purified in this study.

The activities of core enzymes are not confined to BMC-associated functions: aldehyde and alcohol dehydrogenases are utilized in diverse metabolic reactions, and PTAC catalyzes a key biochemical reaction in the process of obtaining energy during fermentation [14]. The concerted functioning of a PTAC and an acetate kinase (Ack) is crucial for ATP generation in the fermentation of pyruvate to acetate (see **Reactions 1** and **2**). Both enzymes are, however, not restricted to fermentative organisms. They can also work in the reverse direction to activate acetate to the CoA-thioester. This occurs, for example, during acetoclastic methanogenesis in the archaeal *Methanosarcina* species [15,16].

**Reaction 1**: acetyl-S-CoA + P_i_ ←→ acetyl phosphate + CoA-SH (PTAC)

**Reaction 2**: acetyl phosphate + ADP ←→ acetate + ATP (Ack)

The canonical PTAC, Pta, is an ancient enzyme found in some eukaryotes [17] and archaea [16], and widespread among the bacteria; 90% of the bacterial genomes in the Integrated Microbial Genomes database [18] contain a gene encoding the PTA_PTB phosphotransacylase (Pfam domain PF01515 [19,20]). Pta has been extensively characterized due to its key role in fermentation [14,21]. More recently, a second type of PTAC without any sequence homology to Pta was identified [4]. This protein, PduL (Pfam domain PF06130), was shown to catalyze the conversion of propionyl-CoA to propionyl-phosphate and is associated with a BMC involved in propanediol utilization, the PDU BMC [4].

Both *pduL* and *pta* genes can be found in genetic loci of functionally distinct BMCs, although the PduL type is much more prevalent, being found in all but one type of metabolosome locus: EUT1 (Table 1) [2]. Furthermore, in the Integrated Microbial Genomes Database [18], 91% of genomes that encode PF06130 also encode genes for shell proteins. As a member of the core biochemical machinery of functionally diverse aldehyde-oxidizing metabolosomes, PduL must have a certain level of substrate plasticity (see Table 1) that is not required of Pta, which has generally been observed to prefer acetyl-CoA [22,23]. PduL from the PDU BMC of *Salmonella enterica* favors propionyl-CoA over acetyl-CoA [4], and it is likely that PduL orthologs in functionally diverse BMCs would have substrate preferences for other CoA derivatives. Another distinctive feature of BMC-associated PduL homologs is an N-terminal encapsulation peptide (EP) that is thought to “target” proteins for encapsulation by the BMC shell [3,24]. EPs are frequently found on BMC-associated proteins and have been shown to interact with shell proteins [25,26]. EPs have also been observed to cause proteins to aggregate [27,28], and this has recently been suggested to be functionally relevant as an initial step in metabolosome assembly, in which a multifunctional protein core is formed, around which the shell assembles [24].

Of the three common metabolosome core enzymes, crystal structures are available for both the alcohol and aldehyde dehydrogenases. In contrast, the structure of PduL, the PTAC found in the vast majority of catabolic BMCs, has not been determined. This is a major gap in our understanding of metabolosome-encapsulated biochemistry and cofactor recycling. Structural information will be essential to working out how the core enzymes and their cofactors assemble and organize within the organelle lumen to enhance catalysis. Moreover, it will be useful for guiding efforts to engineer novel BMC cores for biotechnological applications [1,29,30].

The primary structure of PduL homologs is subdivided into two PF06130 domains, each roughly 80 residues in length. No available protein structures contain the PF06130 domain, and homology searches using the primary structure of PduL do not return any significant results that would allow prediction of the structure. Moreover, the evident novelty of PduL makes its structure interesting in the context of convergent evolution of PTAC function; to-date, only the Pta active site and catalytic mechanism is known [31]. Here we report high-resolution crystal structures of a PduL-type PTAC in both CoA- and phosphate-bound forms, completing our understanding of the structural basis of catalysis by the metabolosome common core enzymes. We propose a catalytic mechanism analogous but yet distinct from the ubiquitous Pta enzyme, highlighting the functional convergence of two enzymes with completely different structures and metal requirements. We also investigate the quaternary structures of three different PduL homologs and situate our findings in the context of organelle biogenesis in functionally diverse BMCs.

## Results

### Structure Determination of PduL

**Fig 2:**
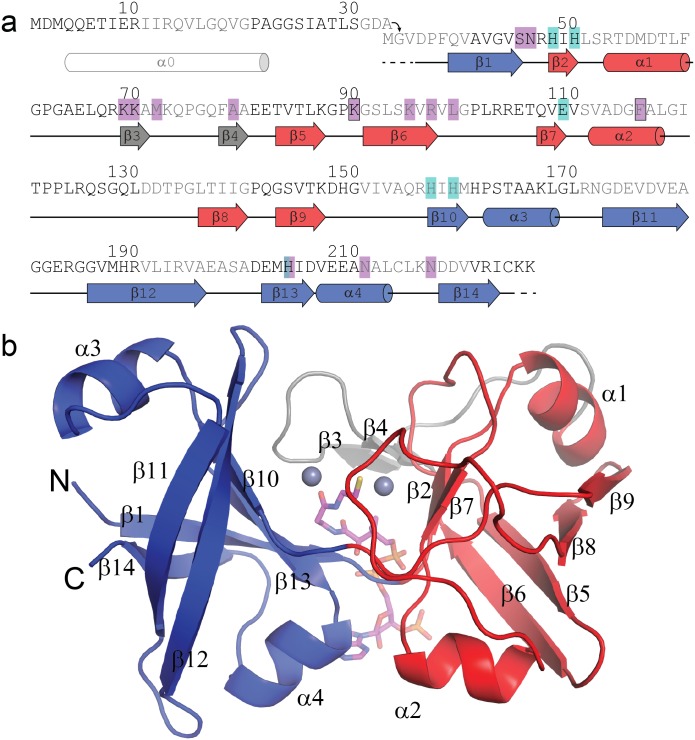
Structural overview of *R*. *palustris* PduL from the *grm3* locus. (**a**) Primary and secondary structure of rPduL (tubes represent α-helices, arrows β-sheets and dashed line residues disordered in the structure. Blocks of ten residues are shaded alternatively black/dark gray. The first 33 amino acids are present only in the wildtype construct and contains the predicted EP alpha helix, α0); the truncated rPduLΔEP that was crystallized begins with M-G-V. Coloring is according to structural domains (domain 1 D36-N46/Q155-C224, blue; loop insertion G61-E81, grey; domain 2 R47-F60/E82-A154, red). Metal coordination residues are highlighted in light blue and CoA contacting residues in magenta, residues contacting the CoA of the other chain are also outlined. (**b**) Cartoon representation of the structure colored by domains and including secondary structure numbering. The N-and C-termini are in close proximity. Coenzyme A is shown in magenta sticks and Zinc (grey) as spheres.

We cloned, expressed, and purified three different PduL homologs from functionally distinct BMCs (Table 1): from the well-studied *pdu* locus in *S*. *enterica* Typhimurium LT2 (sPduL) [3,4], from the recently characterized *pvm* locus in *Planctomyces limnophilus* (pPduL) [32], and from the *grm3* locus in *Rhodopseudomonas palustris* BisB18 (rPduL) [2]. While purifying full-length sPduL, we observed a tendency to aggregation as described previously [4], with a large fraction of the expressed protein found in the insoluble fraction in a white, cake-like pellet. Remarkably, after removing the N-terminal putative EP (27 amino acids), most of the sPduLΔEP protein was in the soluble fraction upon cell lysis. Similar differences in solubility were observed for pPduL and rPduL when comparing EP-truncated forms to the full-length protein, but none were quite as dramatic as for sPduL. We confirmed that all homologs were active (S1a and S1b Fig). Among these, we were only able to obtain diffraction-quality crystals of rPduL after removing the N-terminal putative EP (33 amino acids, also see Fig 2a) (rPduLΔEP). Truncated rPduLΔEP had comparable enzymatic activity to the full-length enzyme (S1a Fig).

We collected a native dataset from rPduLΔEP crystals diffracting to a resolution of 1.54 Å (Table 2). Using a mercury-derivative crystal form diffracting to 1.99 Å (Table 2), we obtained high quality electron density for model building and used the initial model to refine against the native data to Rwork/Rfree values of 18.9/22.1%. There are two PduL molecules in the asymmetric unit of the P2_1_2_1_2_1_ unit cell. We were able to fit all of the primary structure of PduLΔEP into the electron density with the exception of three amino acids at the N-terminus and two amino acids at the C-terminus (Fig 2a); the model is of excellent quality (Table 2). A CoA cofactor as well as two metal ions are clearly resolved in the density (for omit maps of CoA see S2 Fig).

**Table 2:** Data collection and refinement statistics

	PduL native	PduL mercury derivative	PduL phosphate soaked
**Data collection**			
Space group	P 2_1_ 2_1_ 2_1_	P 2_1_ 2_1_ 2_1_	P 2_1_ 2_1_ 2_1_
Cell dimensions			
*a*, *b*, *c* (Å)	57.7, 56.4, 150.4	55.6, 57.7, 150.2	57.1, 58.8, 136.7
*α*, *β*, *γ* (°)	90, 90, 90	90, 90, 90	90, 90, 90
Resolution (Å)	31.4 − 1.54 (1.60 − 1.54)*	35.3 − 1.99 (2.07 − 1.99)	39.2 − 2.10 (2.21 − 2.10)
*R*_merge_	0.169 (1.223)	0.084 (0.299)	0.122 (0.856)
I/σ(I)	12.9 (1.7)	22.1 (7.1)	12.6 (2.0)
Completeness (%)	99.4 (94.4)	99.3 (93.3)	100 (99.9)
Redundancy	13.9 (12.1)	7.2 (7.0)	6.5 (6.1)
**Refinement**			
Resolution (Å)	31.4 − 1.54 (1.60 − 1.54)*		39.2 − 2.10 (2.18 − 2.1)
No. reflections	72,698		27,554
*R*_work/_ *R*_free_ (%)	18.9 (30.7) / 22.1 (34.7)		17.5 (24.2) / 22.6 (30.0)
No. atoms	3,453		3,127
Protein	2,841		2,838
Ligand/ion	100		24
Water	512		265
B-factors	22.8		34.7
Protein	21.5		24.3
Ligand/ion	21.9		40.6
Water	30.3		37.9
R.m.s deviations			
Bond lengths (Å)	0.006		0.013
Bond angles (°)	1.26		1.30
Ramachandran Plot			
favored (%)	99		99
allowed (%)	1		1
disallowed (%)	0		0

*Highest resolution shell is shown in parentheses.

**Fig 3:**
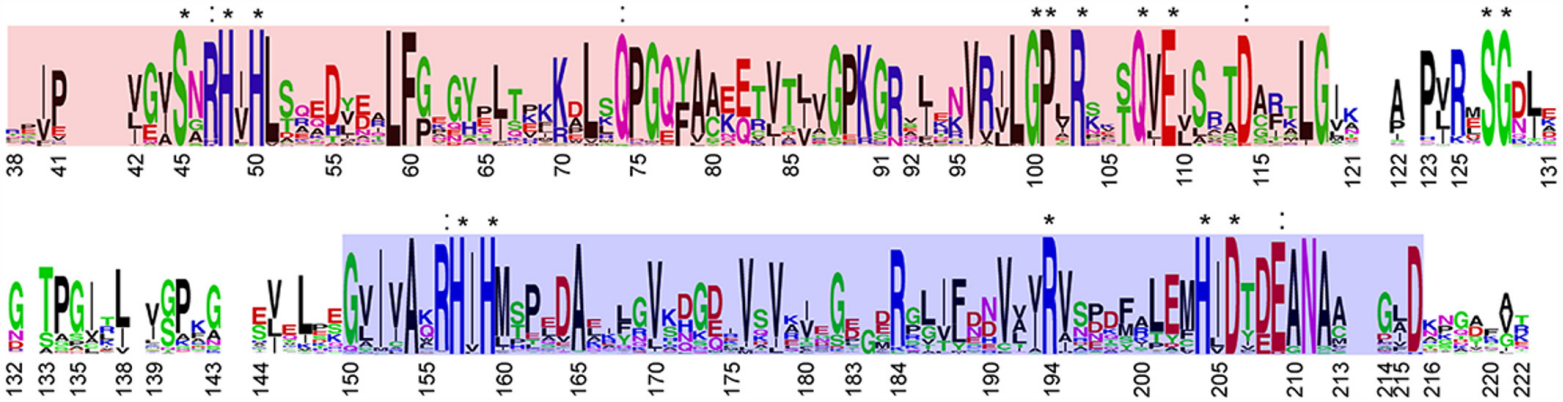
Primary structure conservation of the PduL protein family. Sequence logo calculated from the multiple sequence alignment of PduL homologs (see Materials and Methods), but not including putative EP sequences. Residues 100% conserved across all PduL homologs in our dataset are noted with an asterisk, and residues conserved in over 90% of sequences are noted with a colon. The sequences aligning to the PF06130 domain (determined by BLAST) are highlighted in red and blue. The position numbers shown correspond to the residue numbering of rPduL; note that some positions in the logo represent gaps in the rPduL sequence.

Structurally, PduL consists of two domains (Fig 2, blue/red), each a beta-barrel that is capped on both ends by short α-helices. β-Barrel 1 consists of the N-terminal β strand and β strands from the C-terminal half of the polypeptide chain (β1, β10-β14; residues 37–46 and 155–224). β-Barrel 2 consists mainly of the central segment of primary structure (β2, β5–β9; residues 47–60 and 82–154) (Fig 2, red), but is interrupted by a short two-strand beta sheet (β3-β4, residues 61–81). This β-sheet is involved in contacts between the two domains and forms a lid over the active site. Residues in this region (Gln42, Pro43, Gly44), covering the active site, are strongly conserved (Fig 3). This structural arrangement is completely different from the functionally related Pta, which is composed of two domains, each consisting of a central flat beta sheet with alpha-helices on the top and bottom [31].

**Fig 4:**
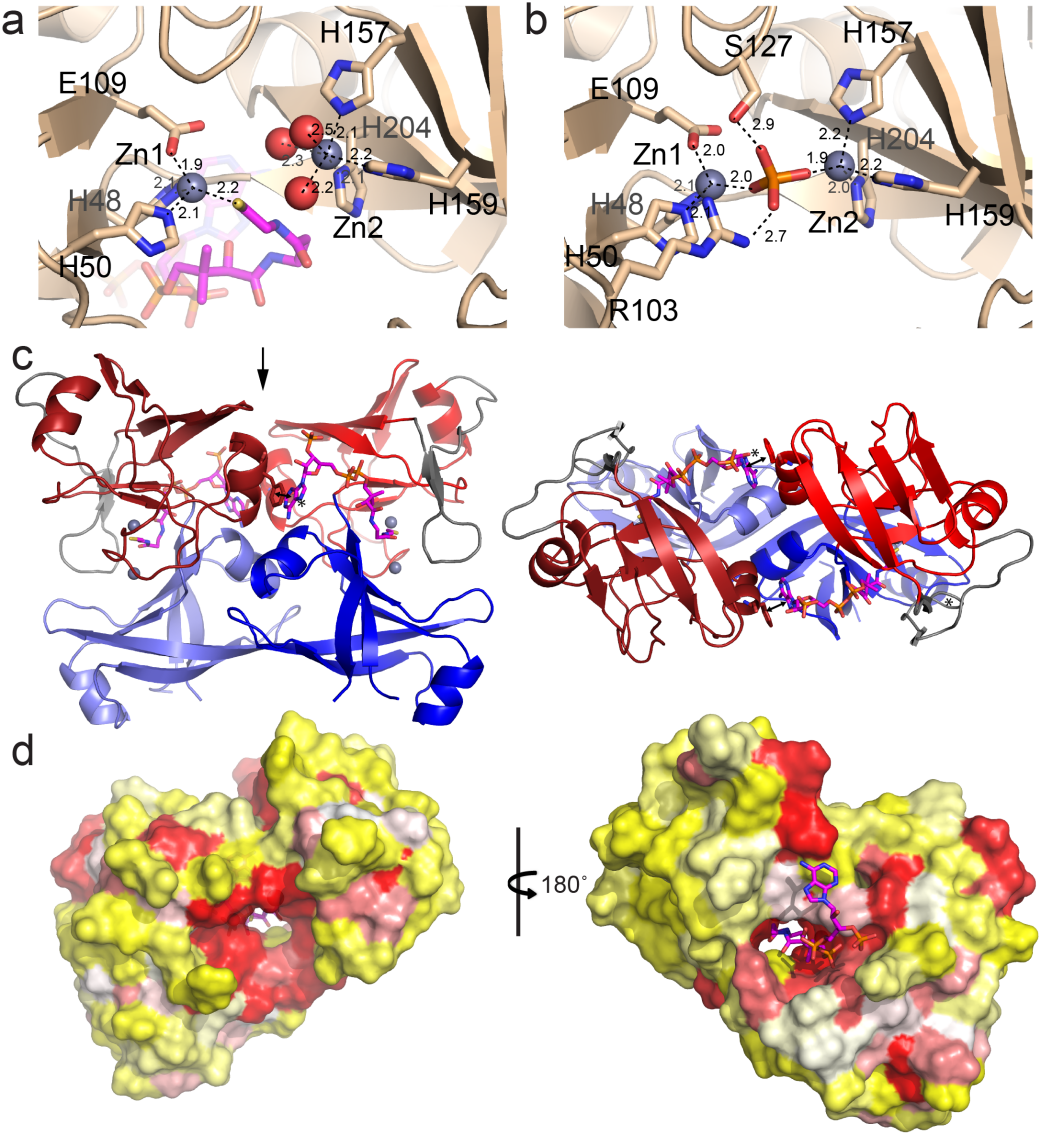
Details of active site, dimeric assembly, and sequence conservation of PduL. (**a**,**b**) Proposed active site of PduL with relevant residues shown as sticks in atom coloring (nitrogen blue, oxygen red, sulfur yellow), zinc as grey colored spheres and coordinating ordered water molecules in red. Distances between atom centers are indicated in Å. (**a**) Coenzyme A containing, (**b**) phosphate-bound structure. (**c**) View of the dimer in the asymmetric unit from the side, domains 1 and 2 colored as in Fig 2 and the two chains differentiated by blue/red versus slate/firebrick. The bottom panel shows a top view down the 2-fold axis as indicated by the arrow in the top panel. The asterisk and double arrow marks the location of the π–π interaction between F116 and the CoA base of the other dimer chain. (**d**) Surface representation of the structure with indicated conservation (red: high, white: intermediate, yellow: low).

**Fig 5:**
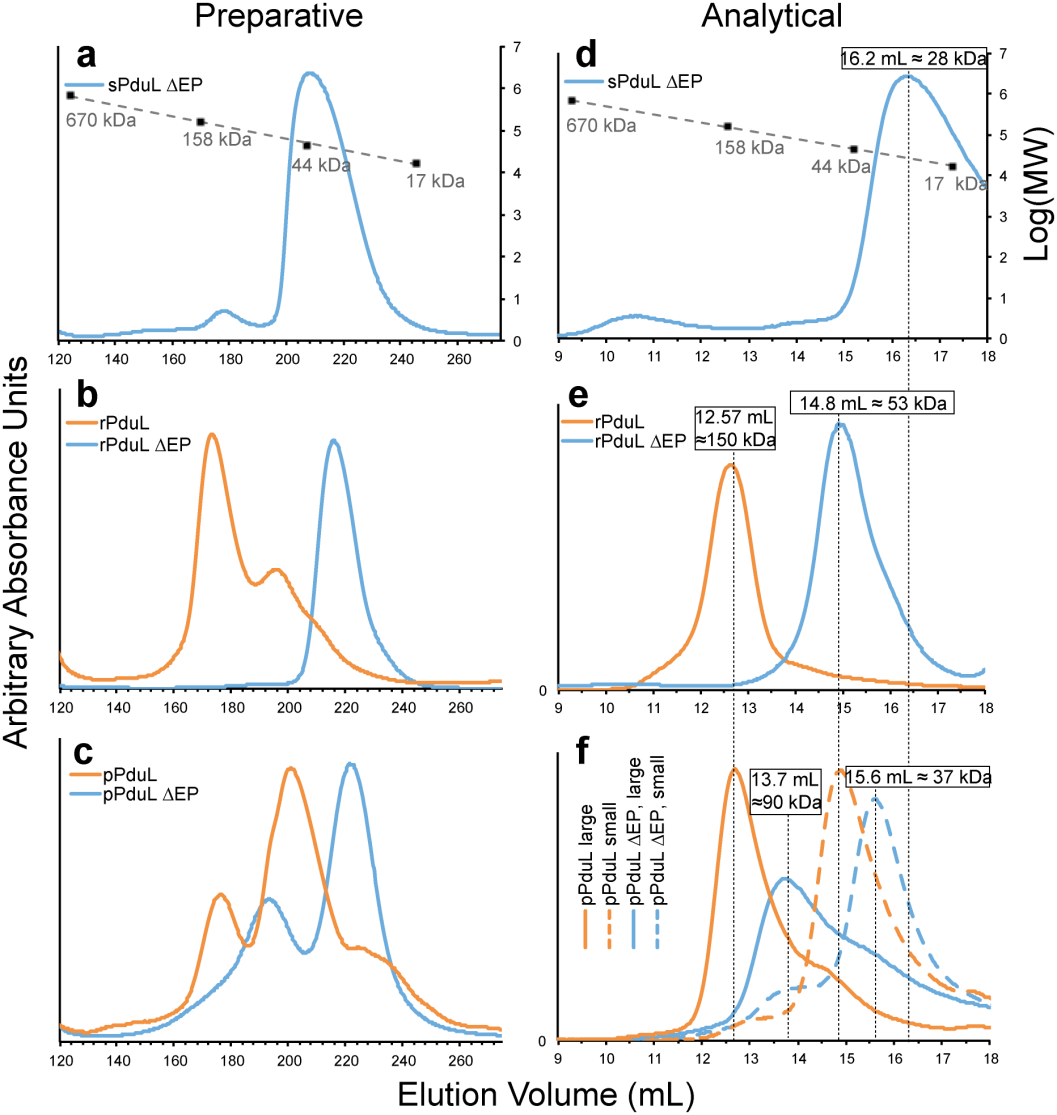
Size exclusion chromatography of PduL homologs. (**a**)–(**c**): Chromatograms of sPduL (**a**), rPduL (**b**), and pPduL (**c**) with (orange) or without (blue) the predicted EP, post-nickel affinity purification, applied over a preparative size exclusion column (see Materials and Methods). (**d**)–(**f**): Chromatograms of sPduL (**d**), rPduL (**e**), and pPduL (**f**) post-preparative size exclusion chromatography with different size fractions separated, applied over an analytical size exclusion column (see Materials and Methods). All chromatograms are cropped to show only the linear range of separation based on standard runs, shown in black squares with a dashed linear trend line. All *y*-axes are arbitrary absorbance units except the right-hand axes for panels (**a**) and (**d**), which is the log_10_(molecular weight) of the standards.

There are two PduL molecules in the asymmetric unit forming a butterfly-shaped dimer (Fig 4c). Consistent with this, results from size exclusion chromatography of rPduLΔEP suggest that it is a dimer in solution (Fig 5e). The interface between the two chains buries 882 Å^2^ per monomer and is mainly formed by α-helices 2 and 4 and parts of β-sheets 12 and 14, as well as a π–π stacking of the adenine moiety of CoA with Phe116 of the adjacent chain (Fig 4c). The folds of the two chains in the asymmetric unit are very similar, superimposing with a rmsd of 0.16 Å over 2,306 aligned atom pairs. The peripheral helices and the short antiparallel β3–4 sheet mediate most of the crystal contacts.

### Active Site Properties

CoA and the metal ions bind between the two domains, presumably in the active site (Figs 2b and 4a). To identify the bound metals, we performed an X-ray fluorescence scan on the crystals at various wavelengths (corresponding to the K-edges of Mn, Fe, Co, Ni, Cu, and Zn). There was a large signal at the zinc edge, and we tested for the presence of zinc by collecting full data sets before and after the Zn K-edge (1.2861 and 1.2822 Å, respectively). The large differences between the anomalous signals confirm the presence of zinc at both metal sites (S3 Fig).

The first zinc ion (Zn1) is in a tetrahedral coordination state with His48, His50, Glu109, and the CoA sulfur (Fig 4a). The second (Zn2) is in octahedral coordination by three conserved histidine residues (His157, His159 and His204) as well as three water molecules (Fig 4a). The nitrogen atom coordinating the zinc is the Nε in each histidine residue, as is typical for this interaction [33]. When the crystals were soaked in a sodium phosphate solution for 2 d prior to data collection, the CoA dissociates, and density for a phosphate molecule is visible at the active site (Table 2, Fig 4b). The phosphate-bound structure aligns well with the CoA-bound structure (0.43 Å rmsd over 2,361 atoms for the monomer, 0.83 Å over 5,259 aligned atoms for the dimer). The phosphate contacts both zinc atoms (Fig 4b) and replaces the coordination by CoA at Zn1; the coordination for Zn2 changes from octahedral with three bound waters to tetrahedral with a phosphate ion as one of the ligands (Fig 4b). Conserved Arg103 seems to be involved in maintaining the phosphate in that position. The two zinc atoms are slightly closer together in the phosphate-bound form (5.8 Å vs 6.3 Å), possibly due to the bridging effect of the phosphate. An additional phosphate molecule is bound at a crystal contact interface, perhaps accounting for the 14 Å shorter c-axis in the phosphate-bound crystal form (Table 2).

### Oligomeric States of PduL Orthologs Are Influenced by the EP

Interestingly, some of the residues important for dimerization of rPduL, particularly Phe116, are poorly conserved across PduL homologs associated with functionally diverse BMCs (Figs 4c and 3), suggesting that they may have alternative oligomeric states. We tested this hypothesis by performing size exclusion chromatography on both full-length and truncated variants (lacking the EP, ΔEP) of sPduL, rPduL, and pPduL. These three homologs are found in functionally distinct BMCs (Table 1). Therefore, they are packaged with different signature enzymes and different ancillary proteins [2]. It has been proposed that the catabolic BMCs may assemble in a core-first manner, with the luminal enzymes (signature enzyme, aldehyde, and alcohol dehydrogenases and the BMC PTAC) forming an initial bolus, or prometabolosome, around which a shell assembles [1]. Given the diversity of signature enzymes (Table 1), it is plausible that PduL orthologs may adopt different oligomeric states that reflect the differences in the proteins being packaged with them in the organelle lumen.

We found that not only did the different orthologs appear to assemble into different oligomeric states, but that quaternary structure was dependent on whether or not the EP was present. Full-length sPduL was unstable in solution—precipitating over time—and eluted throughout the entire volume of a size exclusion column, indicating it was nonspecifically aggregating. However, when the putative EP (residues 1–27) was removed (sPduL ΔEP), the truncated protein was stable and eluted as a single peak (Fig 5a) consistent with the size of a monomer (Fig 5d, blue curve). In contrast, both full-length rPduL and pPduL appeared to exist in two distinct oligomeric states (Fig 5b and 5c respectively, orange curves), one form of the approximate size of a dimer and the second, a higher molecular weight oligomer (~150 kDa). Upon deletion of the putative EP (residues 1–47 for rPduL, and 1–20 for pPduL), there was a distinct change in the elution profiles (Fig 5b and 5c respectively, blue curves). pPduLΔEP eluted as two smaller forms, possibly corresponding to a trimer and a monomer. In contrast, rPduLΔEP eluted as one smaller oligomer, possibly a dimer. We also analyzed purified rPduL and rPduLΔEP by size exclusion chromatography coupled with multiangle light scattering (SEC-MALS) for a complementary approach to assessing oligomeric state. SEC-MALS analysis of rPdulΔEP is consistent with a dimer (as observed in the crystal structure) with a weighted average (M_w_) and number average (M_n_) of the molar mass of 58.4 kDa +/− 11.2% and 58.8 kDa +/− 10.9%, respectively (S4a Fig). rPduL full length runs as M_w_ = 140.3 kDa +/− 1.2% and M_n_ = 140.5 kDa +/− 1.2%. This corresponds to an oligomeric state of six subunits (calculated molecular weight of 144 kDa). Collectively, these data strongly suggest that the N-terminal EP of PduL plays a role in defining the quaternary structure of the protein.

## Discussion

The hallmark attribute of an organelle is that it serves as a discrete subcellular compartment functioning as an isolated microenvironment distinct from the cytosol. In order to create and preserve this microenvironment, the defining barrier (i.e., lipid bilayer membrane or microcompartment shell) must be selectively permeable. The BMC shell not only sequesters specific enzymes but also their cofactors, thereby establishing a private cofactor pool dedicated to the encapsulated reactions. In catabolic BMCs, CoA and NAD^+^ must be continually recycled within the organelle (Fig 1). Homologs of the predominant cofactor utilizer (aldehyde dehydrogenase) and NAD^+^ regenerator (alcohol dehydrogenase) have been structurally characterized, but until now structural information was lacking for PduL, which recycles CoA in the organelle lumen [12,34]. Curiously, while the housekeeping Pta could provide this function, and indeed does so in the case of one type of ethanolamine-utilizing (EUT) BMC [2], the evolutionarily unrelated PduL fulfills this function for the majority of metabolosomes [2,4] using a novel structure and active site for convergent evolution of function.

### The Tertiary Structure of PduL Is Formed by Discontinuous Segments of Primary Structure

The structure of PduL consists of two β-barrel domains capped by short alpha helical segments (Fig 2b). The two domains are structurally very similar (superimposing with a rmsd of 1.34 Å (over 123 out of 320/348 aligned backbone atoms, S5a Fig). However, the amino acid sequences of the two domains are only 16% identical (mainly the RHxH motif, β2 and β10), and 34% similar. Our structure reveals that the two assigned PF06130 domains (Fig 3) do not form structurally discrete units; this reduces the apparent sequence conservation at the level of primary structure. One strand of the domain 1 beta barrel (shown in blue in Fig 2) is contributed by the N-terminus, while the rest of the domain is formed by the residues from the C-terminal half of the protein. When aligned by structure, the β1 strand of the first domain (Fig 2a and 2b, blue) corresponds to the final strand of the second domain (β9), effectively making the domains continuous if the first strand was transplanted to the C-terminus. Refined domain assignment based on our structure should be able to predict domains of PF06130 homologs much more accurately. The closest structural homolog of the PduL barrel domain is a subdomain of a multienzyme complex, the alpha subunit of ethylbenzene dehydrogenase [35] (S5b Fig, rmsd of 2.26 Å over 226 aligned atoms consisting of one beta barrel and one capping helix). In contrast to PduL, there is only one barrel present in ethylbenzene dehydrogenase, and there is no comparable active site arrangement. The PduL signature primary structure, two PF06130 domains, occurs in some multidomain proteins, most of them annotated as Acks, suggesting that PduL may also replace Pta in variants of the phosphotransacetylase-Ack pathway. These PduL homologs lack EPs, and their fusion to Ack may have evolved as a way to facilitate substrate channeling between the two enzymes.

### Implications for Metabolosome Core Assembly

For BMC-encapsulated proteins to properly function together, they must be targeted to the lumen and assemble into an organization that facilitates substrate/product channeling among the different catalytic sites of the signature and core enzymes. The N-terminal extension on PduL homologs may serve both of these functions. The extension shares many features with previously characterized EPs [24,26,36]: it is present only in homologs associated with BMC loci, and it is predicted to form an amphipathic α-helix. Moreover, its removal affects the oligomeric state of the protein. EP-mediated oligomerization has been observed for the signature and core BMC enzymes; for example, full-length propanediol dehydratase and ethanolamine ammonia-lyase (signature enzymes for PDU and EUT BMCs) subunits are also insoluble, but become soluble upon removal of the predicted EP [27,28,11]. sPduL has also previously been reported to localize to inclusion bodies when overexpressed [4]; we show here that this is dependent on the presence of the EP. This propensity of the EP to cause proteins to form complexes (Fig 5) might not be a coincidence, but could be a necessary step in the assembly of BMCs. Structured aggregation of the core enzymes has been proposed to be the initial step in metabolosome assembly [1,37] and is known to be the first step of β-carboxysome biogenesis, where the core enzyme Ribulose Bisphosphate Carboxylase/Oxygenase (RuBisCO) is aggregated by the CcmM protein [37]. Likewise, CsoS2, a protein in the α-carboxysome core, also aggregates when purified and is proposed to facilitate the nucleation and encapsulation of RuBisCO molecules in the lumen of the organelle [36]. Coupled with protein–protein interactions with other luminal components, the aggregation of these enzymes could lead to a densely packed organelle core. This role for EPs in BMC assembly is in addition to their interaction with shell proteins [24–26,36,38].

Moreover, the PduL crystal structures offer a clue as to how required cofactors enter the BMC lumen during assembly. Free CoA and NAD^+^/H could potentially be bound to the enzymes as the core assembles and is encapsulated. However, this raises an issue of stoichiometry: if the ratio of cofactors to core enzymes is too low, then the sequestered metabolism would proceed at suboptimal rates. Our PduL crystals contained CoA that was captured from the *Escherichia coli* cytosol, indicating that the “ground state” of PduL is in the CoA-bound form; this could provide an elegantly simple means of guaranteeing a 1:1 ratio of CoA:PduL within the metabolosome lumen.

### Active Site Identification and Structural Insights into Catalysis

The active site of PduL is formed at the interface of the two structural domains (Fig 2b). As expected, the amino acid sequence conservation is highest in the region around the proposed active site (Fig 4d); highly conserved residues are also involved in CoA binding (Figs 2a and 3, residues Ser45, Lys70, Arg97, Leu99, His204, Asn211). All of the metal-coordinating residues (Fig 2a) are absolutely conserved, implicating them in catalysis or the correct spatial orientation of the substrates. Arg103, which contacts the phosphate (Fig 4b), is present in all PduL homologs. The close resemblance between the structures binding CoA and phosphate likely indicates that no large changes in protein conformation are involved in catalysis, and that our crystal structures are representative of the active form. The native substrate for the forward reaction of rPduL and pPduL, propionyl-CoA, most likely binds to the enzyme in the same way at the observed nucleotide and pantothenic acid moiety, but the propionyl group in the CoA-thioester might point in a different direction. There is a pocket nearby the active site between the well-conserved residues Ser45 and Ala154, which could accommodate the propionyl group (S6 Fig). A homology model of sPduL indicates that the residues making up this pocket and the surrounding active site region are identical to that of rPduL, which is not surprising, because these two homologs presumably have the same propionyl-CoA substrate. The homology model of pPduL also has identical residues making up the pocket, but with a key difference in the vicinity of the active site: Gln77 of rPduL is replaced by a tyrosine (Tyr77) in pPduL. The physiological substrate of pPduL (Table 1) is thought to be lactyl-CoA, which contains an additional hydroxyl group relative to propionyl-CoA. The presence of an aromatic residue at this position may underlie the substrate preference of the PduL enzyme from the *pvm* locus. Indeed, in the majority of PduLs encoded in *pvm* loci, Gln77 is substituted by either a Tyr or Phe, whereas it is typically a Gln or Glu in PduLs in all other BMC types that degrade acetyl- or propionyl-CoA. A comparison of the PduL active site to that of the functionally identical Pta suggests that the two enzymes have distinctly different mechanisms. The catalytic mechanism of Pta involves the abstraction of a thiol hydrogen by an aspartate residue, resulting in the nucleophilic attack of thiolate upon the carbonyl carbon of acetyl-phosphate, oriented by an arginine and stabilized by a serine [31]—there are no metals involved. In contrast, in the rPduL structure, there are no conserved aspartate residues in or around the active site, and the only well-conserved glutamate residue in the active site is involved in coordinating one of the metal ions. These observations strongly suggest that an acidic residue is not directly involved in catalysis by PduL. Instead, the dimetal active site of PduL may create a nucleophile from one of the hydroxyl groups on free phosphate to attack the carbonyl carbon of the thioester bond of an acyl-CoA. In the reverse direction, the metal ion(s) could stabilize the thiolate anion that would attack the carbonyl carbon of an acyl-phosphate; a similar mechanism has been described for phosphatases where hydroxyl groups or hydroxide ions can act as a base when coordinated by a dimetal active site [39].

Our structures provide the foundation for studies to elucidate the details of the catalytic mechanism of PduL. Conserved residues in the active site that may contribute to substrate binding and/or transition state stabilization include Ser127, Arg103, Arg194, Gln107, Gln74, and Gln/Glu77. In the phosphate-bound crystal structure, Ser127 and Arg103 appear to position the phosphate (Fig 4b). Alternatively, Arg103 might act as a base to render the phosphate more nucleophilic. The functional groups of Gln74, Gln/Glu77, and Arg194 are directed away from the active site in both CoA and phosphate-bound crystal structures and do not appear to be involved in hydrogen bonding with these substrates, although they could be important for positioning an acyl-phosphate.

The free CoA-bound form is presumably poised for attack upon an acyl-phosphate, indicating that the enzyme initially binds CoA as opposed to acyl-phosphate. This hypothesis is strengthened by the fact that the CoA-bound crystals were obtained without added CoA, indicating that the protein bound CoA from the *E*. *coli* expression strain and retained it throughout purification and crystallization. The phosphate-bound structure indicates that in the opposite reaction direction phosphate is bound first, and then an acyl-CoA enters. The two high-resolution crystal structures presented here will serve as the foundation for mechanistic studies on this noncanonical PTAC enzyme to determine how the dimetal active site functions to catalyze both forward and reverse reactions.

### Functional, but Not Structural, Convergence of PduL and Pta

PduL and Pta are mechanistically and structurally distinct enzymes that catalyze the same reaction [4], a prime example of evolutionary convergence upon a function. There are several examples of such functional convergence of enzymes, although typically the enzymes have independently evolved similar, or even identical active sites; for example, the carbonic anhydrase family [40,41]. However, apparently less frequent is functional convergence that is supported by distinctly different active sites and accordingly catalytic mechanism, as revealed by comparison of the structures of Pta and PduL. One well-studied example of this is the β-lactamase family of enzymes, in which the active site of Class A and Class C enzymes involve serine-based catalysis, but Class B enzymes are metalloproteins [42,43]. This is not surprising, as β-lactamases are not so widespread among bacteria and therefore would be expected to have evolved independently several times as a defense mechanism against β-lactam antibiotics. However, nearly all bacteria encode Pta, and it is not immediately clear why the Pta/PduL functional convergence should have evolved: it would seem to be evolutionarily more resourceful for the Pta-encoding gene to be duplicated and repurposed for BMCs, as is apparently the case in one type of BMC—EUT1 (Table 1). There could be some intrinsic biochemical difference between the two enzymes that renders PduL a more attractive candidate for encapsulation in a BMC—for example, PduL might be more amenable to tight packaging, or is better suited for the chemical microenvironment formed within the lumen of the BMC, which can be quite different from the cytosol [44,45]. Further biochemical comparison between the two PTACs will likely yield exciting results that could answer this evolutionary question.

### Implications

BMCs are now known to be widespread among the bacteria and are involved in critical segments of both autotrophic and heterotrophic biochemical pathways that confer to the host organism a competitive (metabolic) advantage in select niches. As one of the three common metabolosome core enzymes, the structure of PduL provides a key missing piece to our structural picture of the shared core biochemistry (Fig 1) of functionally diverse catabolic BMCs. We have observed the oligomeric state differences of PduL to correlate with the presence of an EP, providing new insight into the function of this sequence extension in BMC assembly. Moreover, our results suggest a means for Coenzyme A incorporation during metabolosome biogenesis. A detailed understanding of the underlying principles governing the assembly and internal structural organization of BMCs is a requisite for synthetic biologists to design custom nanoreactors that use BMC architectures as a template. Furthermore, given the growing number of metabolosomes implicated in pathogenesis [46–50], the PduL structure will be useful in the development of therapeutics. It is gradually being realized that the metabolic capabilities of a pathogen are also important for virulence, along with the more traditionally cited factors like secretion systems and effector proteins [51]. The fact that PduL is confined almost exclusively to metabolosomes can be used to develop an inhibitor that blocks only PduL and not Pta as a way to selectively disrupt BMC-based metabolism, while not affecting most commensal organisms that require PTAC activity.

## Materials and Methods

### Molecular Cloning

Genes for PduL homologs with and without the EP were amplified via PCR using the primers listed in S1 Table. sPduL was amplified using *S*. *enterica* Typhimurium LT2 genomic DNA, and pPduL and rPduL sequences were codon optimized and synthesized by GenScript with the 6xHis tag. All 5’ primers included EcoRI and BglII restriction sites, and all 3’ primers included a BamHI restriction site to facilitate cloning using the BglBricks strategy. 5’ primers also included the sequence TTTAAGAAGGAGATATACCATG downstream of the restriction sites, serving as a strong ribosome binding site. The 6x polyhistidine tag sequence was added to the 3’ end of the gene using the BglBricks strategy and was subcloned into the pETBb3 vector, a pET21b-based vector modified to be BglBricks compatible.

### Protein Purification, Size Exclusion Chromatography, and Protein Crystallization

*E*. *coli* BL21(DE3) expression strains containing the relevant PduL construct in the pETBb3 vector were grown overnight at 37°C in standard LB medium and then used to inoculate 1L of standard LB medium in 2.8 L Fernbach flasks at a 1:100 dilution, which were then incubated at 37°C shaking at 150 rpm, until the culture reached an OD600 of 0.8–1.0, at which point cultures were induced with 200 μM IPTG (isopropylthio-β-D-galactoside) and incubated at 20°C for 18 h, shaking at 150 rpm. Cells were centrifuged at 5,000 xg for 15 min, and cell pellets were frozen at –20°C.

For protein purifications, cell pellets from 1–3 L cultures were resuspended in 20–30 ml buffer A (50 mM Tris-HCl pH 7.4, 300 mM NaCl) and lysed using a French pressure cell at 20,000 lb/in^2^. The resulting cell lysate was centrifuged at 15,000 xg. 30 mM imidazole was added to the supernatant that was then applied to a 5 mL HisTrap column (GE Healthcare Bio-Sciences, Pittsburgh, PA). Protein was eluted off the column using a gradient of buffer A from 0 mM to 500 mM imidazole over 20 column volumes. Fractions corresponding to PduL were pooled and concentrated using Amicon Ultra Centrifugal filters (EMD Millipore, Billerica, MA) to a volume of no more than 2.5 mL. The protein sample was then applied to a HiLoad 26/60 Superdex 200 preparative size exclusion column (GE Healthcare Bio-Sciences, Pittsburgh, PA) and eluted with buffer B (20 mM Tris pH 7.4, 50 mM NaCl). Where applicable, fractions corresponding to different oligomeric states were pooled separately, leaving one or two fractions in between to prevent cross contamination. Pooled fractions were concentrated to 1–20 mg/mL protein as determined by the Bradford method [52] prior to applying on a Superdex 200 10/300 GL analytical size exclusion column (GE Healthcare Bio-Sciences, Pittsburgh, PA). Size standards used were Thyroglobulin 670 kDa, γ-globulin 158 kDa, Ovalbumin 44 kDa, and Myoglobin 17 kDa. For light scattering, the proteins were measured in a Protein Solutions Dynapro dynamic light scattering instrument with an acquisition time of 5 s, averaging 10 acquisitions at a constant temperature of 25°C. The radii were calculated assuming a globular particle shape.

Size exclusion chromatography coupled with SEC-MALS was performed on full-length rPduL and rPduL-ΔEP similar to Luzi et al. 2015 [53]. A Wyatt DAWN Heleos-II 18-angle light scattering instrument was used in tandem with a GE AKTA pure FPLC with built in UV detector, and a Wyatt Optilab T-Rex refractive index detector. Detector 16 of the DAWN Heleos-II was replaced with a Wyatt Dynapro Nanostar QELS detector for dynamic light scattering. A GE Superdex S200 10/300 GL column was used, with 125–100 μl of protein sample at 1 mg/ml concentration injected, and the column run at 0.5 ml/min in 20 mM Tris, 50 mM NaCl, pH 7.4.

Each detector of the DAWN-Heleos-II was plotted with the Zimm model in the Wyatt ASTRA software to calculate the molar mass. The molar mass was measured at each collected data point across the peaks at ~1 point per 8 μl eluent. Both the M_w_ and M_n_ of the molar mass calculations, as well as percent deviations, were also determined using Wyatt software program ASTRA.

For preparing protein for crystallography, expression cells were grown as above, except were induced with 50 μM IPTG. Harvested cells were resuspended in buffer B and lysed using a French Press. Cleared lysate was applied on a 5 ml HisTrap HP column (GE Healthcare) and washed with buffer A containing 20 mM imidazole. Pdul-His was eluted with 2 CV buffer B containing 300 mM imidazole, concentrated and then applied on a HiLoad 26/60 Superdex 200 (GE Healthcare) column equilibrated in buffer B for final cleanup. Protein was then concentrated to 20–30 mg/ml for crystallization. Crystals were obtained from sitting drop experiments at 22°C, mixing 3 μl of protein solution with 3 μl of reservoir solution containing 39%–35% MPD. Crystals were flash frozen in liquid nitrogen after being adding 5 μl of a reservoir solution. For heavy atom derivatives, 0.2 μl of 100 mM Thiomerosal (Hampton Research) was added to the crystallization drop 36 h prior to freezing. For phosphate soaks, 5 μl reservoir and 1.5 μl 200 mM sodium phosphate solution (pH 7.0) were added 2 d prior to flash freezing.

### PTAC Activity Assay

Enzyme reactions were performed in a 2 mL cuvette containing 50 mM Tris-HCl pH 7.5, 0.2 mM 5,5'-dithiobis-2-nitrobenzoic acid (DTNB; Ellman’s reagent), 0.1 mM acyl-CoA, and 0.5 μg purified PTAC, unless otherwise noted. To initiate the reaction, 5 mM NaH_2_PO_4_ was added, the cuvette was inverted to mix, and the absorbance at 412 nm was measured every 2 s over the course of four minutes in a Nanodrop 2000c, in the cuvette holder. 14,150 M^-1^cm^-1^ was used as the extinction coefficient of DTNB to determine the specific activity.

### PduL Sequence Analysis

A multiple sequence alignment of 228 PduL sequences associated with BMCs [2] and 20 PduL sequences not associated with BMCs was constructed using MUSCLE [54]. PduL sequences associated with BMCs were determined from Dataset S1 of Reference [2], and those not associated with BMCs were determined by searching for genomes that encoded PF06130 but not PF03319 nor PF00936 in the IMG database [18]. The multiple sequence alignment was visualized in Jalview [55], and the nonconserved N- and C-terminal amino acids were deleted. This trimmed alignment was used to build the sequence logo using WebLogo [56].

### Diffraction Data Collection, Structure Determination and Visualization

Diffraction data were collected at the Advanced Light Source at Lawrence Berkeley National Laboratory beamline 5.0.2 (100 K, 1.0000 Å wavelength for native data, 1.0093 Å for mercury derivative, 1.2861 Å for Zn pre-edge and 1.2822 Å for Zn peak). Diffraction data were integrated with XDS [57] and scaled with SCALA (CCP4 [58]). The structure of PduL was solved using phenix.autosol [59], which found 11 heavy atom sites and produced density suitable for automatic model building. The model was refined with phenix.refine [59], with refinement alternating with model building using 2Fo-Fc and Fo-Fc maps visualized in COOT [60]. Statistics for diffraction data collection, structure determination and refinement are summarized in Table 2. Figures were prepared using pymol (www.pymol.org) and Raster3D [61].

### Homology Modeling

Models of *S*. *enterica* Typhimurium LT2 and *P*. *limnophilus* PduL were generated with Modeller using the align2d and model-default scripts [62].

## Supporting Information

S1 Data Data for S1a Fig. (XLSX)

S2 Data Data for S2b Fig. (XLSX)

S1 Fig Enzymatic activity of PduL homologs. (**a**) sPduLΔEP, rPduL hexamer and rPduLΔEP, (**b**) pPduL hexamer and dimer—pPduL experiments were performed with 8 μg purified protein.
(TIFF)

S2 Fig Simulated annealing omit map of Coenzyme A region (a) and the active site (b). Fofc density in green at 1.8 rmsd from a simulated annealing refinement run omitting Coenzyme A, Zn atoms, and water molecules.
(TIF)

S3 Fig Metal identification by differential anomalous signals. Anomalous map density contoured at 0.025 e^-^/Å^3^ in the vicinity of the metal sites for data collected at 1.2822 Å (red, Zn peak) and 1.2861 Å (yellow map, Zn pre-edge) identifies the metals as zinc based on the large decrease of signal when collecting the data above the Zn edge.
(TIF)

S4 Fig SEC-MALS analysis using Wyatt ASTRA software indicating the calculated mass of the peaks for (A) rPduLΔEP and (B) rPduL full length. (TIF)

S5 Fig Analysis of the structural domains of PduL. (**a**) Structural alignment of domain 1 (red) and domain 2 (blue), linker region colored grey. (**b**) Structural alignment of domain 1 of PduL with a subdomain (residues 872–936 and 967–976 of chain A) of the ethylbenzene dehydrogenase from *Aromatoleum aromaticum* (pdb ID 2IVF).
(TIF)

S6 Fig Potential propionyl binding pocket in PduL. The area around conserved residues A154 and S45 forms a potential binding pocket for the propionyl group (shaded ellipsoid).
(TIF)

S1 Table Primers used in this study. (DOCX)

## Abbreviations

Ack: acetate kinase
BMC: Bacterial Microcompartment
EP: encapsulation peptide
EUT: ethanolamine-utilizing
M_n_: number average
M_w_: weighted average
PDU: propanediol-utilizing
Pta: phosphotransacylase
PTAC: phosphotransacylase
RuBisCO: Ribulose Bisphosphate Carboxylase/Oxygenase
SEC-MALS: multiangle light scattering
