# Supplementary figures and images for: The Structural Basis of Coenzyme A Recycling in a Bacterial Organelle
Source: PLoS Biol. 2016 Mar 9;14(3):e1002399. doi: 10.1371/journal.pbio.1002399 (PMC4784909; doi:10.1371/journal.pbio.1002399)

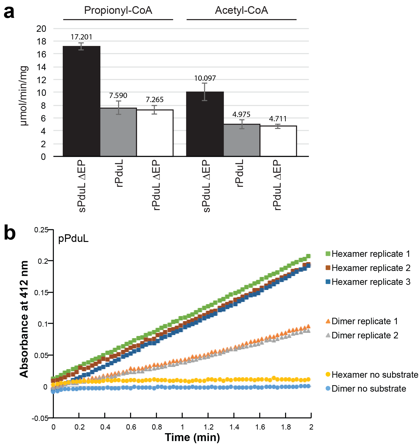

Supplement: S1 Fig — (a) sPduLΔEP, rPduL hexamer and rPduLΔEP, (b) pPduL hexamer and dimer—pPduL experiments were performed with 8 μg purified protein. (TIFF) [file pbio.1002399.s003.tiff]

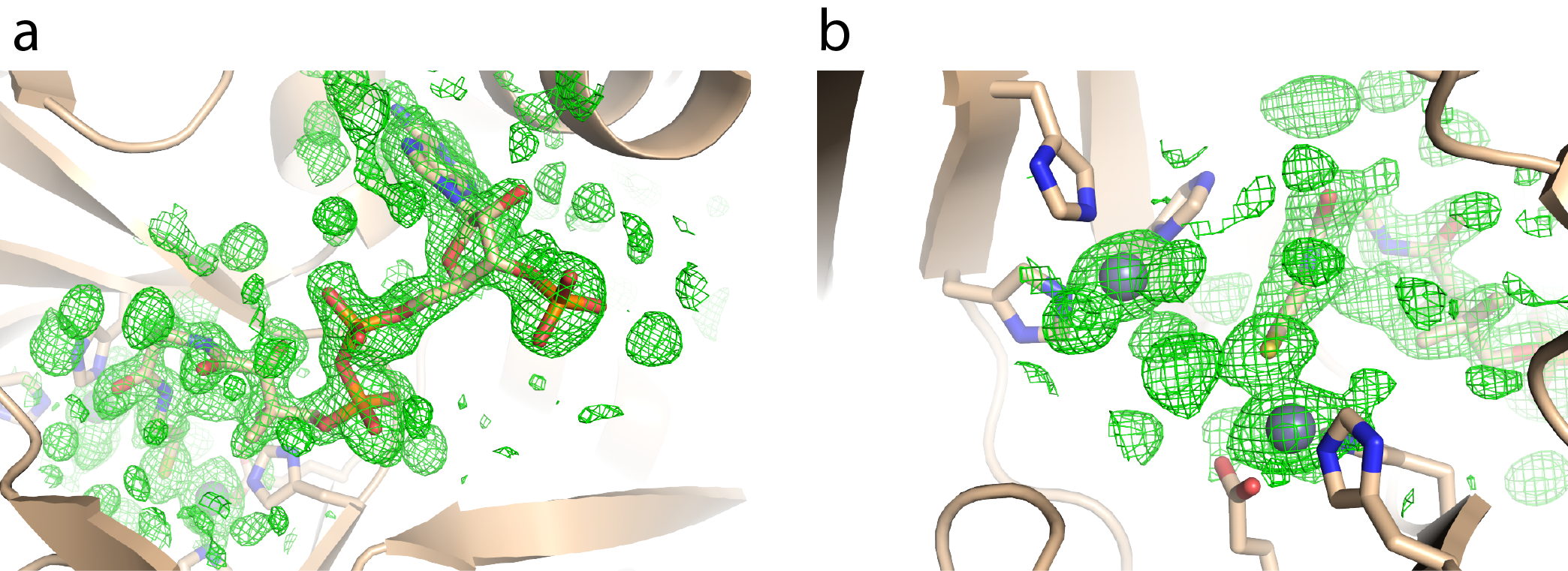

Supplement: S2 Fig — Fofc density in green at 1.8 rmsd from a simulated annealing refinement run omitting Coenzyme A, Zn atoms, and water molecules. (TIF) [file pbio.1002399.s004.tif]

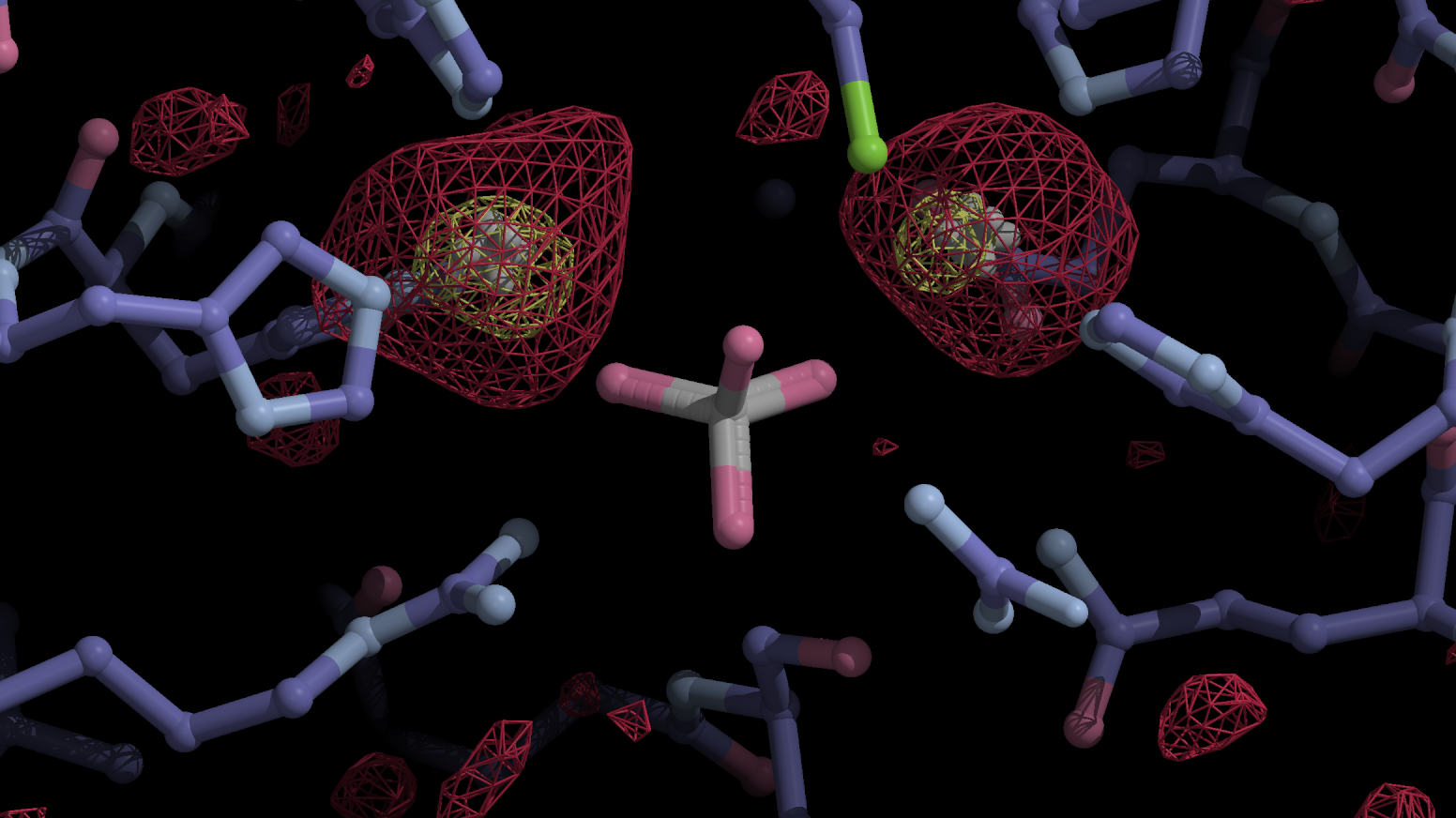

Supplement: S3 Fig — Anomalous map density contoured at 0.025 e-/Å3 in the vicinity of the metal sites for data collected at 1.2822 Å (red, Zn peak) and 1.2861 Å (yellow map, Zn pre-edge) identifies the metals as zinc based on the large decrease of signal when collecting the data above the Zn edge. (TIF) [file pbio.1002399.s005.tif]

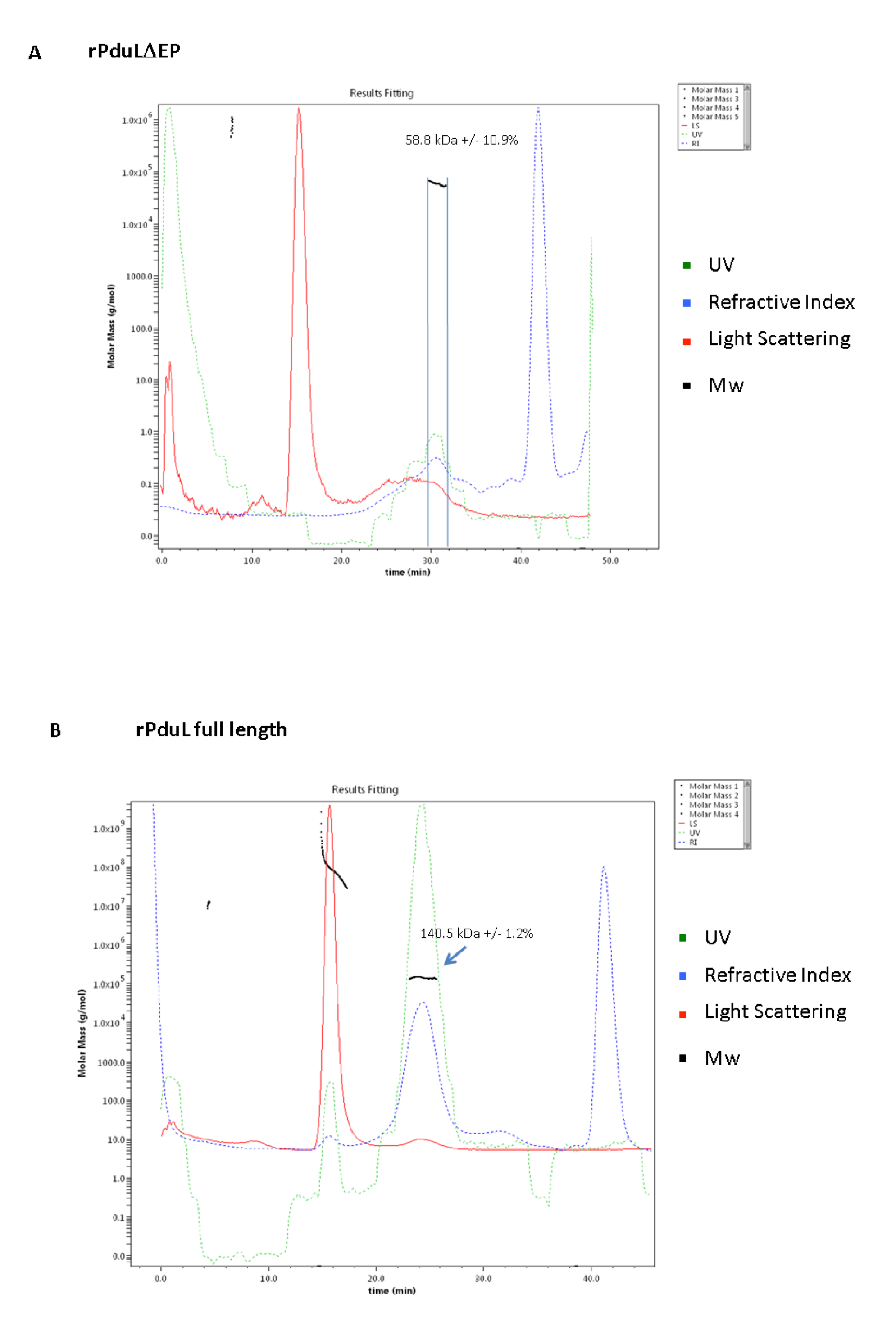

Supplement: S4 Fig — (TIF) [file pbio.1002399.s006.tif]

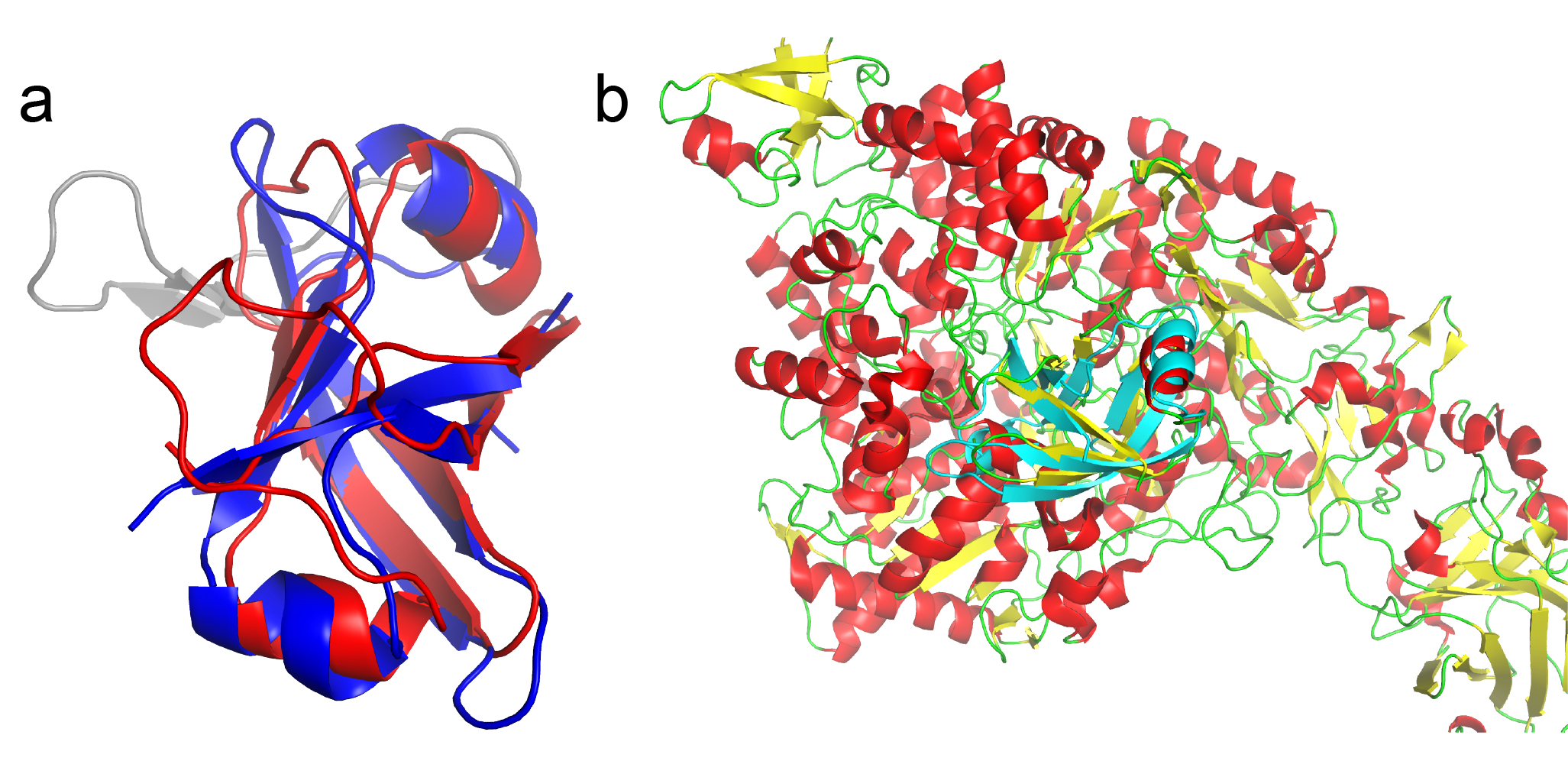

Supplement: S5 Fig — (a) Structural alignment of domain 1 (red) and domain 2 (blue), linker region colored grey. (b) Structural alignment of domain 1 of PduL with a subdomain (residues 872–936 and 967–976 of chain A) of the ethylbenzene dehydrogenase from Aromatoleum aromaticum (pdb ID 2IVF). (TIF) [file pbio.1002399.s007.tif]

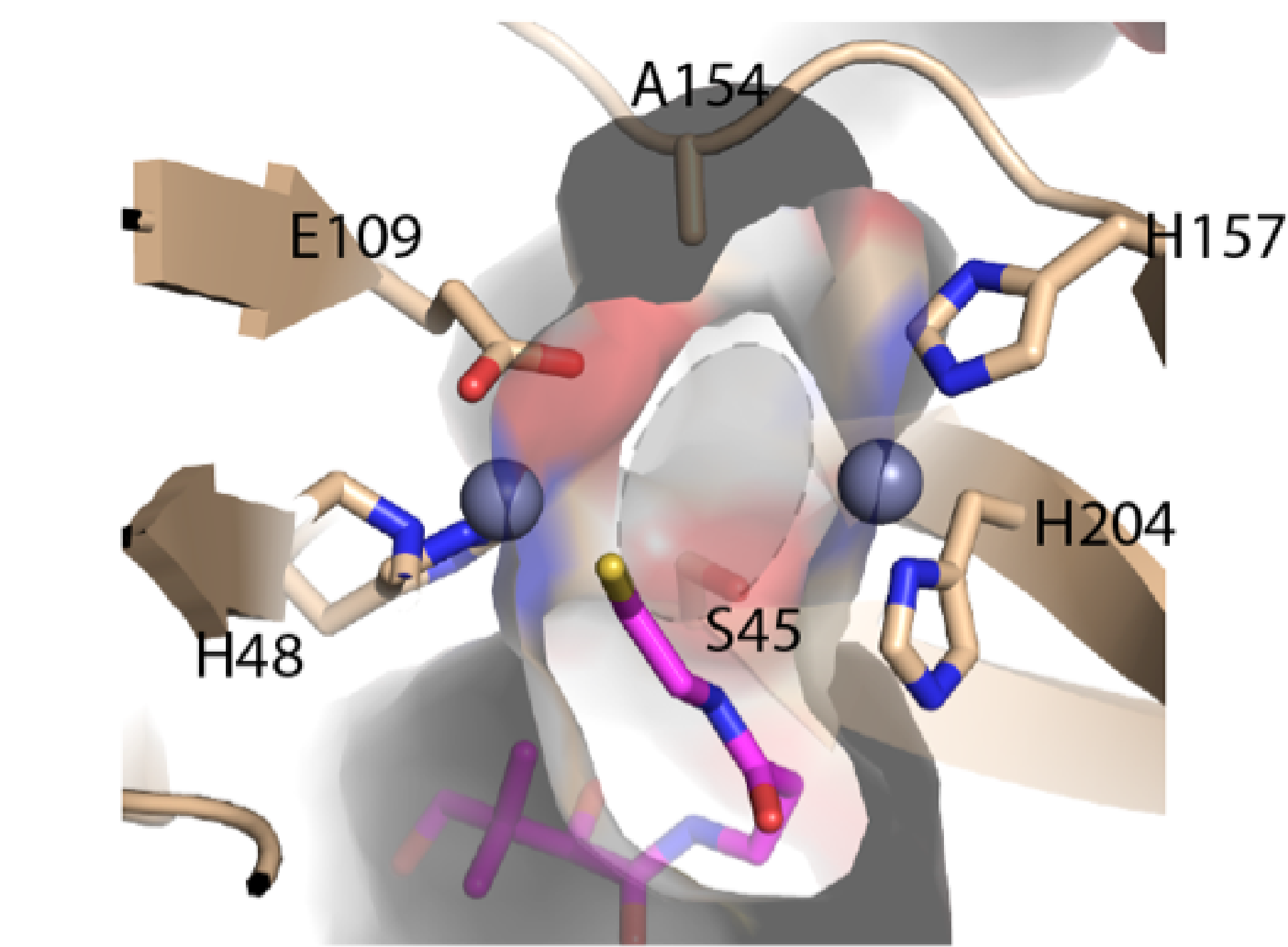

Supplement: S6 Fig — The area around conserved residues A154 and S45 forms a potential binding pocket for the propionyl group (shaded ellipsoid). (TIF) [file pbio.1002399.s008.tif]
