# Supplementary material for: The Structural Basis of Coenzyme A Recycling in a Bacterial Organelle
Source: PLoS Biol. 2016 Mar 9;14(3):e1002399. doi: 10.1371/journal.pbio.1002399 (PMC4784909; doi:10.1371/journal.pbio.1002399)
Supplement: S1 Table — (DOCX) [file pbio.1002399.s009.docx]

**S1 Table. Primers used in this study.**

| **Primer number** | **Sequence** | **Description** |
| --- | --- | --- |
| OE257 | atagaattcatgagatcttttaagaaggagatataccatggataaagagcttctgcaatc | sPduL forward |
| OE259 | tataggatcctcgcgggcctaccagc | sPduL reverse |
| OE384 | atagaattcatgagatcttttaagaaggagatataccatgattccgctgggcgtctc | sPduL ΔEP forward |
| OE385 | atagaattcatgagatcttttaagaaggagatataccatgctggttgtgaatatttcagcacg | pPduL ΔEP forward |
| OE423 | tatagaattcatgagatcttttaagaaggagatataccatgggcgtggacccttttcaag | rPduL ΔEP forward |
| OE323 | tataggatccttagtggtggtggtggtggtg | 6xHis reverse |
